# 4,4'Dimethoxychalcone: a natural flavonoid that promotes health through autophagy-dependent and -independent effects

**DOI:** 10.1080/15548627.2019.1632623

**Published:** 2019-06-28

**Authors:** Andreas Zimmermann, Katharina Kainz, Sebastian J. Hofer, Maria A. Bauer, Sabrina Schroeder, Jörn Dengjel, Federico Pietrocola, Oliver Kepp, Christoph Ruckenstuhl, Tobias Eisenberg, Stephan J. Sigrist, Frank Madeo, Didac Carmona-Gutierrez, Guido Kroemer

**Affiliations:** a Institute of Molecular Biosciences, NAWI Graz, University of Graz, Graz, Austria; b Division of Endocrinology and Diabetology, Department of Internal Medicine, Medical University of Graz, Graz, Austria; c BioTechMed Graz, Graz, Austria; d Department of Biology, Université de Fribourg, Fribourg, Switzerland; e Institute for Research in Biomedicine; Barcelona, Spain; f Equipe 11 labellisée Ligue contre le Cancer, Centre de Recherche des Cordeliers, INSERM U 1138, Paris, France; g Metabolomics and Cell Biology Platforms, Gustave Roussy Comprehensive Cancer Center, Villejuif, France; h Université Paris Descartes, Sorbonne Paris Cité, Paris, France; i Central Lab Gracia, NAWI Graz, University of Graz, Graz, Austria; j Institute for Biology/Genetics, Freie Universität Berlin, Berlin, Germany; k Pôle de Biologie, Hôpital Européen Georges Pompidou, Paris, France; l Karolinska Institute, Department of Women’s and Children’s Health, Karolinska University Hospital, Stockholm, Sweden; m Suzhou Institute for Systems Biology, Chinese Academy of Sciences, Suzhou, China; n Université Pierre et Marie Curie, Paris, France; o BioHealth Graz, Graz, Austria

**Keywords:** Cardioprotection, flavonoid, GATA, liver protection, longevity

## Abstract

The age-induced deterioration of the organism results in detrimental and ultimately lethal pathologies. The process of aging itself involves a plethora of different mechanisms that should be subverted concurrently to delay and/or prevent age-related maladies. We have identified a natural compound, 4,4ʹ-dimethoxychalcone (DMC), which promotes longevity in yeast, worms and flies, and protects mice from heart injury and liver toxicity. Interestingly, both the DMC-mediated lifespan extension and the cardioprotection depend on macroautophagy/autophagy whereas hepatoprotection does not. DMC induces autophagy by inhibiting specific GATA transcription factors (TFs), independently of the TORC1 kinase pathway. The autophagy-independent beneficial effects of DMC might involve its antioxidative properties. DMC treatment results in a phylogenetically conserved, systemic impact on the metabolome, which is most prominently characterized by changes in cellular amino acid composition. Altogether, DMC exerts multiple, geroprotective effects by igniting distinct pathways, and thus represents a potential pharmacological agent that delays aging through multipronged effects.

The standard approach to fight age-related pathologies (e.g. metabolic syndrome, neurodegenerative disorders, neoplastic and cardiovascular disease) consists in treating each of them separately. However, this concept undervalues the complex interconnection between these diseases and underestimates the pliability of the aging process as their common denominator. An alternative path for prevention or therapy may involve interventions with intrinsic multi-target effects. In that respect, the permanent or periodic reduction of calorie intake (without malnutrition) has proven effective in promoting health and lifespan effects that are triggered by different (possibly intertwined) mechanisms. Among them are the induction of protective autophagy, the reduction of oxidative stress, anti-inflammatory effects, and other systemic metabolic adaptations. However, most individuals are incapable of observing a lifestyle that involves dietary constraints. This has prompted the search for drugs that potentially revert the age-associated decline by activating (some of) the beneficial metabolic effects of caloric restriction. To date, only a few substances with such potential have been found, including nicotinamide adenine dinucleotide precursors, metformin, resveratrol, rapamycin and spermidine.

**Figure 1.**
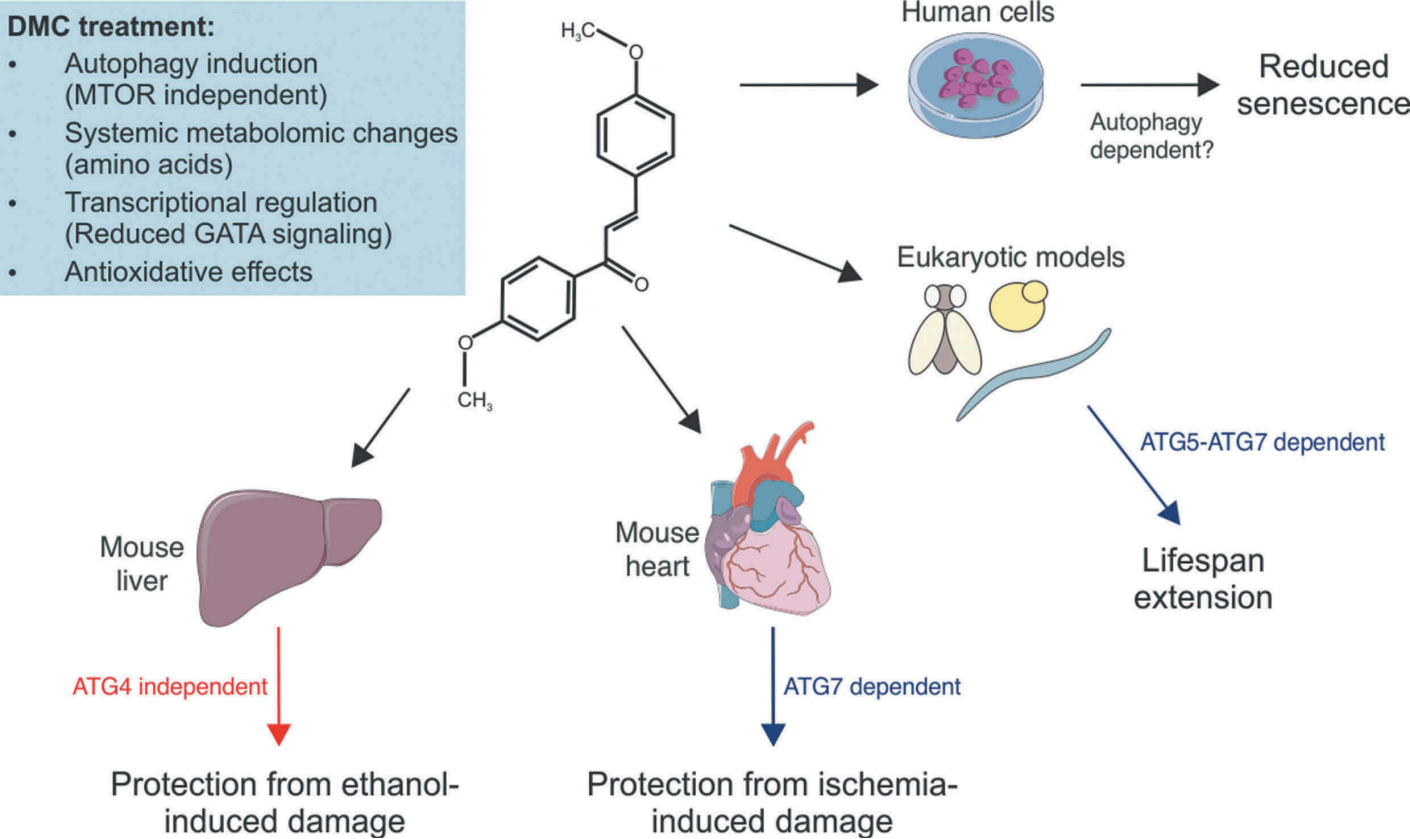
4,4ʹ-dimethoxychalcone (DMC) promotes health through autophagy-dependent and -independent effects. DMC treatment results in a characteristic intracellular response with a number of beneficial effects across species. Autophagy is causal for lifespan extension in yeast, nematodes and fruit flies as well as for cardioprotection in mice. *Per contra*, the protective effects of DMC in the liver, where autophagy is also induced upon DMC treatment, do not depend on *Atg4b*, suggesting pathways alternative to canonical autophagy. Illustrations adapted from Servier Medical Art, licensed under a CC-BY 3.0 Unported License.

In our recent work, we identified yet another natural substance, the flavonoid 4,4′-dimethoxychalcone (DMC), that possesses the capacity to slow aging [1]. DMC treatment extends the lifespan of yeast, worms and flies (three established aging models) and slows senescence, the age-related permanent arrest of the cell cycle, in human cell cultures. Additionally, we demonstrated cardio- and hepatoprotective effects of intraperitoneally injected DMC in mice. DMC significantly reduces the infarction area in the heart tissue caused by prolonged myocardial ischemia. Moreover, DMC dampens the ethanol-induced increase of circulating serum GPT/alanine aminotransferase activity, which is a biomarker of liver damage. In sum, these results show that the health-promoting effects of DMC are phylogenetically conserved across distinct species.

Importantly, DMC increases autophagic flux in all tested organisms, and this autophagy enhancement is causally linked to most of its protective effects. Upon disruption (or silencing) of specific autophagy-related (*ATG*) genes, DMC loses its ability to increase lifespan in yeast, worms and flies. Moreover, DMC fails to protect mice from myocardial ischemia if *Atg7* is conditionally ablated in the heart muscle (Figure 1). However, there are 2 exceptions to this dependency on autophagy. (1) In yeast, the DMC effects only require autophagy at later time points but not at the early stage of chronological aging. An explanation might be that DMC – like many other flavonoids – has antioxidant properties, which may act independently from autophagy. In yeast, DMC was only supplied once at the start of the experiment, and the initial protection may rely on the acute and direct reduction of oxidative stress. (2) The hepatoprotective effects of DMC in mice are maintained in whole body *Atg4b* knockout animals (Figure 1); nevertheless, DMC does significantly promote autophagic flux in the liver. While our data imply that this induction is merely correlative, we did not explore whether it may correspond to a non-canonical form of autophagy that would not require *Atg4b* and/or whether other *Atg4* isoforms may be involved. Should that be the case, a causal relationship between DMC’s pro-autophagic effect in the liver and hepatoprotection could still be possible. Thus, further experiments addressing this question are warranted.

Irrespective of additional non-autophagic mechanisms, the main protective effects of DMC do depend on autophagy. Thereby, specific GATA transcription factors (TFs) seem crucial. In yeast, deletion of *GLN3* (but not other GATA TF genes) precludes DMC cytoprotection. Similarly, silencing of specific GATA TFs in nematodes (ELT-1) and human cell culture (most prominently GATA2) abolish the DMC-mediated effects. Our data suggest that DMC might function via inhibition of these specific GATA TFs, because DMC treatment strongly reduces Gln3 activity in yeast, and results in a similar metabolomic shift as *GLN3* disruption. In line, *gln3* deletion mutants manifest both increased lifespan and elevated autophagy levels *per se* during chronological aging.

Beyond the mode of action of DMC, our work poses a broader question: are GATA TFs general determinants of aging? Exploring the relevance of GATA TFs in more complex eukaryotic aging will require dissecting which of the multiple GATA family members contributes to aging, perhaps acting in a tissue-specific fashion, a possibility that might render difficult the exploration of this system. In line with a functional role of GATA TFs during aging, it has been recently suggested that in *Drosophila*, the GATA factor srp (serpent) is involved in a regulatory hub that links dietary essential amino acids, dietary restriction and longevity.

A further mechanistic finding is that DMC operates independently of TORC1 kinase, a master regulator of autophagy, the inhibition of which has been extensively associated with longevity. *Vice versa*, rapamycin (a specific TORC1 inhibitor) does not require GATA TFs to induce autophagy and to extend lifespan, at least in yeast. This opens the possibility to combine TORC1-dependent and -independent interventions with the hope of obtaining even more profound geroprotective effects. In fact, as a proof of principle, we found that DMC and rapamycin exert additive cytoprotective effects in yeast.

Finally, our data reveal that DMC has a major impact on amino acid metabolism. The proteome of DMC-treated yeast is characterized by a significant downregulation of proteins involved in amino acid regulation, but also – although to a lower extent – of proteins connected to carboxylic acid, organic acid, amine and nitrogen components. Similarly, metabolomic analysis of DMC-treated yeast cells as well as of cardiac and hepatic tissues from mice subjected to intraperitoneal DMC treatment show decreased levels of most amino acids. Although these systemic metabolic changes are only correlative, they may reflect a general reprogramming that is essential for DMC-mediated effects, especially in view of the known influence of amino acid metabolism on aging and diverse age-related diseases. Of note, amino acid regulation is strongly determined via transcription, and other transcriptional regulators (beyond GATA factors) might be involved in the metabolic impact of DMC.

In sum, DMC seems to mediate its beneficial effects through multiple routes, including antioxidative and pro-autophagic mechanisms as well as other (possibly connected) systemic metabolic adaptations. This aligns well with the concept that caloric restriction-like interventions should involve multi-target effects. In that respect, DMC (or derivatives thereof) may indeed become a valid pharmacological path for the prevention or treatment of age-related diseases.
